# Memory's Malleability: Its Role in Shaping Collective Memory and Social Identity

**DOI:** 10.3389/fpsyg.2012.00257

**Published:** 2012-07-23

**Authors:** Adam D. Brown, Nicole Kouri, William Hirst

**Affiliations:** ^1^Posttraumatic Stress Disorder Research Program, Department of Psychiatry, New York University School of MedicineNew York, NY, USA; ^2^Cognitive Science Laboratory, Department of Psychology, New School for Social ResearchNew York, NY, USA

If anything has been learned about memory, it is that it is fragile and error prone (Schacter, 2001; Loftus, 2005). Far from being a verbatim record of the past, memory is well understood as a reconstructive process replete with distortions, and at times, gross inaccuracies. Although often associated with negative consequences (Wells and Olson, 2003; McNally and Geraerts, 2009) there is growing evidence to suggest that memory's imperfections may also be a virtue (Schacter, 2012; Schacter et al., 2011). The reconstructive nature of memory is believed to provide greater cognitive flexibility (Schacter and Addis, 2007), underlie mental time travel (Schacter et al., 2008; Szpunar, 2010), and support the construction and maintenance of self-identity and life-stories (Greenwald, 1980; Markus and Nurius, 1986; Bruner, 1991; Baerger and McAdams, 1999; Conway and Pleydell-Pearce, 2000; Wilson and Ross, 2001). We argue here that memory's malleability benefits more than just the self – the same attitudes, schemata, and social and physical environments that render an individual's memory unique can also transform initially disparate memories into shared recollections. It is our proposition that autobiographical memories are simultaneously reconstructed to be distinct from that of another person and converge with it as a result of social interactions. Through this convergence, emerges collective memory that will in turn establish a collective identity and promote sociality. Our aim here is to bridge the gap between individual and collective memory by discussing several lines of research elucidating the processes by which the malleability of memory promotes the formation of shared memories.

## The Psychological Study of Collective Memory

Collective memories are a community's shared renderings of the past that help shape its collective identity (Halbwachs, 1950). From this perspective, they are the collective variant of autobiographical memories, which are individually held memories that help shape personal identity. The identity-constructing function of collective memories implies that not all shared memories are collective memories. That is, a memory can only be considered collective if it is widely shared and if it helps to define and bind together a group (Assmann, 1995). For example, Americans are, to a degree, Americans because they possess shared renderings of the past, and Americans differ from Russians, in part, because the two hold different shared memories for similar historical events. For instance, these two nations remember War World II differently. Americans tend to remember D-Day as being the most important battle of the war; Russians remember the most important battle as the Battle of Leningrad (Wertsch, 2002). Their different memories help shape the way Russians and Americans see their place in the world and how they conceive of themselves as a nation.

Whereas psychologists have largely remained on the sidelines of collective memory research, the last few years has evidenced a growing body of literature relevant to the psychological study of collective memory (Cuc et al., 2006; Barnier and Sutton, 2008; Stone et al., 2010; Coman and Hirst, 2012). It seeks to examine the cognitive mechanisms underlying how individual memories emerge, spread, and become shared across a community. These cognitive mechanisms often involve memory distortions, but as we shall see, these distortions are often shared across community memories, and as a result lead to shared memories.

Probably the best understood mechanisms for creating shared memories has been discussed in a various ways since Bartlett (1932) first introduce the notion of schema. He suggested that shared memories may be formed through social interactions because community members, whom are raised together, attend the same school, read the same books, and generally share many of the same experiences, will possess similar schemata, and in turn will shape the way community members remember their past. Take, for example, Hastrof and Cantril's (1954) study of Dartmouth and Princeton students’ memories of a critical football match between the two schools; within group memories were similar, whereas across the two populations, the memories were dissimilar.

Despite these results, it is not always the case that a shared culture and shared schemata will dominate the shaping of one's memory. Community members achieve their individuality, in part, because they possess unshared attitudes and schemata. The discrepancies, as such, can lead members of the same community to remember a shared event quite differently. Paradoxically, individually distinct memories can still become shared over time. What makes us claim that memory is well-designed for the formation of collective memory is that there are a variety of mechanisms and processes that will lead to mnemonic convergence, in spite of the dissonance that exists among rememberers.

These mechanisms can shape and reshape memory through a variety of means. We focus here on conversational interactions. Although memory may have a number of functions (Bluck, 2003), the communicative function of memory may be uniquely human (Pillemer, 1992), and talking about the past is a pervasive part of everyday life (Hirst and Echterhoff, 2012). Pasupathi et al. (2009) found that 62% of events recorded in diary entries had already been discussed the evening after they had occurred. Similarly, Harber and Cohen (2005) found that after 33 students visited a morgue on a field trip, 881 people knew of the visit after three conversational exchanges had taken place. Furthermore, eyewitnesses tend to talk to other co-witnesses after witnessing incidents (Skagerberg and Wright, 2008).

When investigating how conversations shape memory studies to date have focused primarily on the impact a speaker has on a listener's memory. Whereas Echterhoff, Higgins, and others have focused on the reflexive influence a speaker can have on reshaping his or her memory, leading to mnemonic convergence between speaker and listener, a so-called *shared reality* (Hirst and Echterhoff, 2008; Echterhoff et al., 2009), speakers can have a unilateral influence on a listener's memory, leading to a similar shared reality.

## Social Contagion

Through acts of social remembering individuals become vulnerable to incorporating details about the past that they did not actually experience. That is, conversations can serve as a mechanism enabling the spread of a memory from one person to another. This process is often referred to as social contagion. Social contagion can be traced back to the classic work of Loftus and colleagues (Echterhoff et al., 2005; Loftus, 2005). Although Loftus did not frame her work in terms of social contagion research, these experiments and others (Loftus, 2005) have consistently demonstrated that social interactions (e.g., what an experimenter says to a participant) can be an effective means for implanting false memories. For example, in these studies participants were asked to view a series of slides depicting a traffic accident. After the initial viewing, an experimenter provided them with additional information describing the accident, information that at times contradicted the content in the original images. After the post-event information was given to the subject, participants were asked to recall what they had seen. Across numerous studies Loftus and colleagues have demonstrated the ability to implant false memories for a wide range of events including getting lost in a grocery store, knocking over a wedding cake, and seeing Hanna–Barbara cartoon figures at Disneyland (Loftus, 2005).

Although the implantation of false memories often occurred from exposure to a social stimuli, studies directly examining how the effects of social interactions on memory have shown that social interactions are particularly effective methods for shaping memories. For example, Meade and Roediger (2002) asked participants to view a complex image. Afterward, a confederate discussed the image with the participant, providing false information relating to the original image. Post-discussion, participants were asked to individually recollect what they had originally seen. Although subjects were more likely to incorporate related, novel information into their recollections, even unrelated/unexpected implanted content was included and accepted as a valid memory. Wright et al. (2000) found that two people unknowingly integrated their individual memories of slightly different pictures with that of his or her peer. Cuc et al. (2006) went even further – groups of four were asked to first individually recollect the story they had just read, and then to discuss it with each other, and found across several studies that conversations are an effective means for transforming how different version of the past can converge into a more uniform memory.

The relationship the listener has with the speaker impacts what is transmitted within the conversation. Individuals are prone to conformity; they are not bent on providing novel information to a group recollection. The rememberers are following the conversational maxim – *say no more than is necessary* (Grice, 1975). What goes unsaid during the initial stages of conversation will be less likely to be included in the final shared memory. In essence, conversation and by extension memory transmission is sometimes a democratic process – frequency of participation determines how much influence one has over the group recollection. Put simply, the more one dominates a conversation the greater impact they will have on shaping the group's collective memory (Cuc et al., 2006), a conversational role referred to as the “dominant narrator.” In fact, dominant narrators appear to be more effective in shaping collective memory than perceived experts (Brown et al., 2009).

## Socially Shared Retrieval-Induced Forgetting

Collective memory is inherently selective (Rajaram and Pereira-Pasarin, 2010; Hirst and Echterhoff, 2012). When people recall the past some details are retrieved while other fail to enter into conversation. The consequence of those items not retrieved has become of increasing interest in understanding how distinct memories become increasingly similar across individuals. Hirst and colleagues (Stone et al., 2012) have conducted studies applying the retrieval-induced forgetting (RIF) paradigm (Anderson et al., 1994) to social interactions. RIF in individuals consistently show that recalling an item inhibits the accessibility of categorically related information. In other words, retrieving a piece of information, a part of memory, makes it harder to remember unrecalled related information than if the individual had not retrieved any aspect of that memory at all. Modifying this paradigm to social interactions, similar patterns were found. That is, when people converse about the past evidence of RIF patterns emerge not only for the person doing the recalling but for the person listening to the speaker as well (socially shared RIF, SS-RIF; for a review see Stone et al., 2012). Hirst and colleagues posit that this occurs when the listeners concurrently retrieves with the speaker. SS-RIF has been found in free flowing conversations (Cuc et al., 2007), flashbulb memories (Coman et al., 2009), and in clinical populations (Brown et al., 2012). Interestingly, this effect was found even when speakers and listeners possessed similar, but not identical memories. Coman et al. (2009) asked individuals, unknown to each other, who had been living in New York City on 9/11 to recall their memories of that day. The results showed unmentioned details related to what was recalled became not only harder for the speaker to later remember but also in the listener as well, even though the speaker did not share these exact memories. These findings suggest that when people collectively recall the past, the act of retrieval has the potential to induce forgetting across individuals in similar ways, and like social contagion can also be an effective means for creating collective memories.

Are there certain conditions that increase the probability that a speaker can induce forgetting in a listener? A recent study by Barber and Mather (2012) found that RIF in speakers and listeners was greater when both participants were of the same gender, whereas neither the valence of the memory exchange (i.e., neutral versus negative) nor the age cohort of the participants had significant influence on the rate of forgetting. Barber and Mather's (2012) findings suggest that affiliation between speaker and listener may enhance forgetting. Emotion may also play a role. Brown et al. (2012) asked combat veterans with and without PTSD to study and selectively recall either trauma or neutral stimuli. Although equal levels of forgetting were found for neutral information, individuals with PTSD exhibited greater levels of induced forgetting, individually and socially, for trauma-related stimuli. Future studies will benefit from elucidating more clearly the conditions when social forgetting will and will not occur.

## Conclusion

This paper has illustrated the capacity for memory's malleability to facilitate sociality and transform individual memories into shared, and subsequently collective memories. The transformation of individual memory into collective memory can be seen as an emergent and recursive system(s). We argue that the mechanisms that guide mnemonic convergence are in it of themselves social mediators. The porous nature of memory helps an individual maneuver through a social world that consists of an aggregate of autobiographical memories, and in so doing the individual as such engenders collective remembrance. Coman and Hirst (2012) found that mnemonic influences, such as social contagion and SS-RIF, are transitive and strengthen as they propagate. The plurality of the process is inevitable given the multiple environments individuals exist within. What begins as a dyadic exchange, results in a cohesive network, that is sustained by a multiplicity of convergences within and between groups.
